# Jk Dna Gaga Motifs Are Required for Local Nucleosome Remodeling and Vk-jk Recombination

**DOI:** 10.21203/rs.3.rs-8340154/v1

**Published:** 2025-12-30

**Authors:** Marcus Clark, Margaret Veselits, Kaitlin McLean, Nathaniel Wright, Michael Okoreeh, Jacob Veselits, Mark Maienschein-Cline, Malay Mandal

**Affiliations:** University of Chicago; University of Chicago; Department of Medicine, Section of Rheumatology and Gwen Knapp Center for Lupus and Immunology Research, University of Chicago; University of Chicago; University of Chicago; University of Chicago; University of Illinois at Chicago; University of Chicago

## Abstract

Immunoreceptor gene recombination requires complementary 12 bp and 23 bp recombination signal sequences (RSSs). In addition, at RSSs where RAG proteins assemble, recombination centers must be accessible yet have a proximate nucleosome decorated with H3K4me3 for RAG2 recruitment. In *Drosophila*, DNA GAGA motifs play an important role in nucleosome positioning. Herein, we report that 5’ to each functional Jk 23bp-RSS is a DNA GAGA motif conserved across mammalian species. In mice, the GAGA motif 5’ to Jk1 regulated local RSS accessibility and 5’ nucleosome placement. Furthermore, it was required for efficient Vk-Jk1 recombination. Murine Jk3 is nonfunctional, having mutations in both RSS and GAGA motifs. Adding back both GAGA and RSS motifs restored Jk3 RSS accessibility and Vk-Jk3 recombination. In contrast, the RSS alone did not significantly restore accessibility or recombination. Additionally, examination of genomic sequences revealed similar GAGA motifs 5’ to each JH 23bp-RSS. Our studies indicate that in developing B cells, specific GAGA-dependent mechanisms regulate nucleosome positioning and accessibility at Jk for efficient Vk-Jk recombination. Furthermore, our data suggest an expanded definition of recombination center-associated RSSs in B cells to include a 5’ GAGA sequence that dictates the local epigenetic state required for gene recombination.

## Summary

Recombination center assembly requires a specific epigenetic topology at recombination signal sequences. Herein, we report that conserved GAGA motifs 5’ to each Jk segment are required for establishing this epigenetic topology and subsequent local gene recombination.

## Introduction

B lymphocytes produce a vast repertoire of antibodies to protect against a myriad of pathogens. This diversity is largely achieved through stochastic V(D)J recombination of the immunoglobulin (Ig) genes^1, 2^. Ig gene cleavage is mediated by proteins encoded by the two recombinase activating genes, *RAG1* and *RAG2*^3, 4^. RAG protein-induced double strand breaks (DSBs) occur at specific recombination signal sequences (RSSs) containing highly conserved nonamer and heptamer motifs, separated by 12 or 23 base pairs. The “12/23 Rule” dictates that recombination must occur between one RSS containing a 12 bp spacer and one RSS containing a 23 bp spacer^5, 6, 7^. At *Igk*, each Vk gene segment has a 3’ 12 bp RSS and each functional Jk gene segment has a 5’ 23 bp RSS. The RAG complex assembles at Jk RSSs to form recombination centers to which the Vk gene segments are recruited^1, 2, 8, 9, 10^.

While gene recombination is necessary for antigen receptor diversity, any mistargeting of the RAG complex risks genomic translocations and malignant transformation^11, 12, 13^. Indeed, throughout the genome there are cryptic RSSs (cRSSs), which can be cleaved by the RAG complex and lead to genomic instability^14, 15, 16^. To mitigate this risk, mechanisms have evolved to ensure the fidelity of the RAG complex.

For example, the exquisite temporal, cell cycle, and tissue-specific regulation of RAG expression restricts gene recombination to specific lymphocyte developmental states^2, 3, 4, 17, 18^. Furthermore, RAG recruitment to DNA is regulated epigenetically as RAG2 binds nucleosomes decorated with histone H3 lysine 4 trimethylation (H3K4me3) via its plant homeodomain (PHD) finger domain^19, 20, 21^. These data suggest that *in vivo*, efficient RAG recruitment and recombination require the local presence of nucleosomes^22^.

Paradoxically, when bound *in vitro* to nucleosomes, RSSs are resistant to cleavage by RAG^23, 24^. Furthermore, nucleosomes preferentially bind RSS-containing DNA sequences^25^. These data indicate that nucleosomes bearing H3K4me3 required for RAG2 recruitment must be precisely positioned at Jk to both recruit RAG2 yet allow RAG1-mediated RSS cleavage^2^. Indeed, this necessary topology is enforced by the epigenetic reader BRWD1, which remodels local nucleosome structure to both place a nucleosome 5’ to each Jk RSS and to ensure each RSS is accessible^26^.

Genome-wide, there is a striking association between BRWD1-dependent nucleosome remodeling and the presence of GA-repeats in DNA (“GAGA motifs”)^26^. In *Drosophila*, the epigenetic modifier GAGA Factor (GAF) plays a critical role in the expression of homeotic genes^27, 28^. GAF is a pioneer transcription factor that binds to GA-rich DNA sequences, with a GAGAG pentamer being the canonical consensus sequence^29^. However, GAF can bind a minimal GAG trinucleotide motif^30^. GAF works with chromatin remodeling complexes in an ATP-dependent manner to slide or evict nucleosomes located in proximity to GAGA motifs^31, 32, 33^. However, the role of GAGA motifs in modulating nucleosome positioning in mammals is unknown.

Herein, we report that conserved GAGA motifs located 5’ to each Jk RSS play a crucial role in establishing the nucleosome architecture required for efficient *Igk* recombination. Mutation and add-back experiments demonstrate that GAGA motifs are both necessary and sufficient to dictate local epigenetic structure around Jk 23bp-RSSs and enable Vk-Jk recombination in small pre-B cells. These data suggest that nucleosome positioning is a critical component of the epigenetic regulation that both enables and restricts cleavage to canonical RSSs in B lymphocytes.

## Results

### Jk1 GAGA motif is necessary for efficient Vk-Jk1 recombination

**Figure 1:**
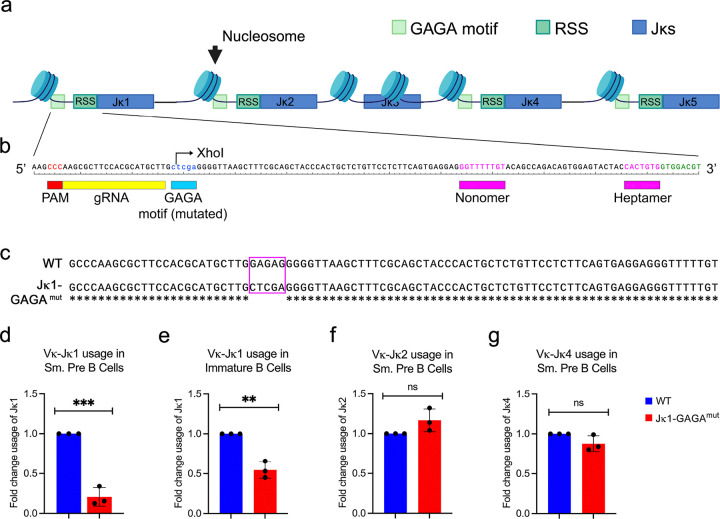
Jk1 5’ GAGA region required for *Igk* recombination. **(A)** A simplified representation of the Jk locus, which consists of 5 gene segments (blue), of which 4 functional gene segments (Jk1, Jk2, Jk4, and Jk5) are preceded by an RSS (teal) and a GAGA motif domain (green). Jk3 is a nonfunctional segment and lacks both 5’ elements. **(B)** A CRISPR guide was designed to mutate the GAGA motif without disturbing the RSS. **(C)** The results of sequencing from the CRISPR-Cas9-edited founder mouse line. **(D-E)** Quantitative RT-PCR for the Vk-Jk1 recombination product in flow-sorted small pre-B (D) and immature (E) B cells isolated from WT and *Jk1-GAGA*^*mut*^ mice (each point represents separate mouse). **(F-G)** Quantitative RT-PCR for the Vk-Jk2 (F) and Vk-Jk4 (G) recombination products in flow-sorted small pre-B cells isolated from WT and *Jk1-GAGA*^*mut*^ mice. (n = 3 mice, unpaired t-test, *p <0.05, **p <0.01).

Examination of nucleotide sequences at the Jk locus revealed that each functional Jk gene segment was preceded by a GAGAG DNA sequence (“GAGA motif”) located an average of approximately 80 base pairs (bp) 5’ to each RSS (Figure 1A). Interestingly, the non-functional Jk3 gene segment was not associated with a GAGAG motif. Furthermore, a comparison of the sequence 5’ to the *Igk* Jk1 segment across various vertebrate species indicated that this GAGA motif was conserved (**Supplementary Figure 1**). In most species examined, it was located within 80 bp of the Jk1 RSS, while in others it was located approximately 350 bp upstream. We hypothesized that if this motif is important for nucleosome positioning and *Igk* recombination, its removal should result in reduced usage of the corresponding Jk in the expressed *Igk* repertoire.

To test this prediction, we used CRISPR-CAS9 gene editing to create a mouse model in which the Jk1 upstream GAGA motif was mutated (Figure 1B). A guide RNA was designed to both target the Jk1 GAGA motif and introduce a *Xho1* site to facilitate screening. The guide RNA was incubated with CAS9 protein and injected in single cell mouse embryos as a ribonucleoprotein (RNP) complex. By genomic PCR, digestion with *Xho1* and then sequencing, we identified a founder in which the GAGAG motif was mutated to CTCGA (Figure 1C). This founder was bred to homozygosity (*Jk1-GAGA*^*mut*^*)*.

*Igk* recombination is initiated in small pre-B cells^1, 34^. Therefore, to determine how loss of the Jk1 5’ GAGA motif region affected Jk1 usage in developing B cells, we sorted small pre-B (B220^low^CD19^+^CD43^−^IgM^−^FSC^low^) and immature B cells (B220^low^CD19^+^CD43^−^IgM^+^) from wild type (WT) and *Jk1-GAGA*^*mut*^ mice (**Supplementary Figure 2**). RNA was isolated, reverse transcribed (RT), and quantitative PCR (qPCR) for Vk-Jk1 recombination products performed^26^. In small pre-B cells, *Jk1-GAGA*^*mut*^ cells had severely decreased Vk-Jk1 recombination (approximately 5-fold) and this defect persisted into immature B cells (Figures 1D–E). However, RT-PCR for Vk-Jk2 and Vk-Jk4 recombination products revealed normal frequencies (Figures 1F–G). These data indicate that the Jk1 GAGA motif specifically regulates Vk-Jk1 recombination.

### Jk1 GAGA motif dictates local chromatin structure

**Figure 2:**
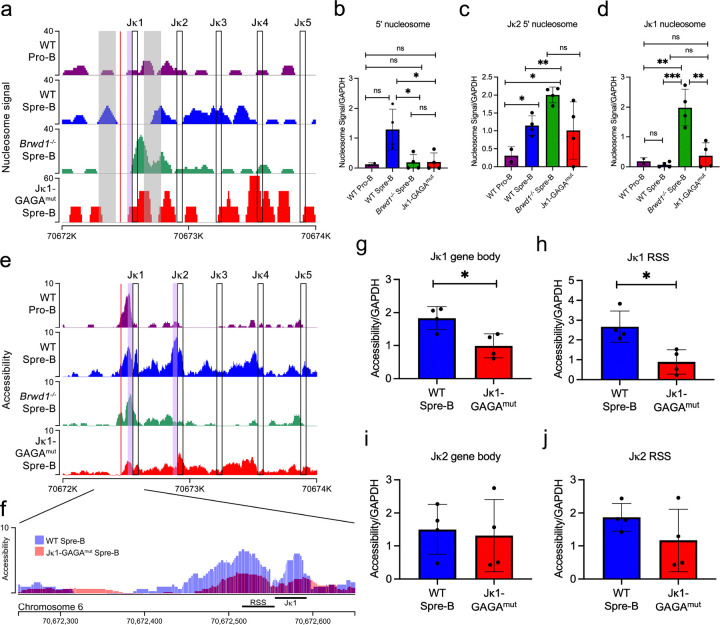
Jk1 GAGA motif required for 5’ nucleosome placement and RSS accessibly. **(A)** Nucleosome positioning at the Jk locus in WT pro B, WT small pre-B, *Brwd1*^*−/−*^ small pre-B and *Jk1-GAGA*^*mut*^ small pre-B cells. Nucleosome signal represents the difference in normalized density between the simulated signal and background data, with signal defined from read pairs with large insert sizes and background defined from read pairs with short insert sizes. The open boxes indicate the position of the respective gene bodies, the lilac shaded region indicates the Jk1 RSS and the red line indicates the Jk1 GAGA motif. Position of 5’ nucleosomes are indicated by grey boxes. Data are representative of two independent experiments for the WT pro B and four independent experiments for the WT small pre-B, *Brwd1*^*−/−*^ small pre-B and *Jk1-GAGA*^*mut*^ small pre-B cells. **(B)** Quantification of 5’ nucleosome signal at Jk1. **(C)** Quantification of 5’ nucleosome at Jk2. **(D)** Quantification of nucleosome signal at the Jk1 gene segment plus RSS. **(E-F)** Examples of accessibility at the Jk locus as measured by ATAC-seq. The y axis represents tags per million reads. Data representative of two independent experiments for the WT pro B and four independent experiments for the WT small pre-B, *Brwd1*^*−/−*^ small pre-B and *Jk1-GAGA*^*mut*^ small pre-B. **(G)** Quantification of chromatin accessibility at the Jk1 gene body. **(H)** Quantification of chromatin accessibility at the Jk1 RSS. **(I)** Quantification of chromatin accessibility at the Jk2 gene body. **(J)** Quantification of chromatin accessibility at the Jk2 RSS. (each point is from a separate mouse)(unpaired t-test, *p <0.05, **p <0.01, ***p<0.001).

In WT small pre-B cells, the Jk RSSs are accessible and flanked by nucleosomes predicted to recruit RAG2^19, 20, 21, 26^. This chromatin state is dictated by BRWD1 ^26^. Therefore, we first sought to determine whether deletion of the 5’ Jk1 GAGA motif domain affected Jk1 nucleosome density. For this, we performed ATAC (assay for transposase-accessible chromatin with sequencing) with paired-end sequencing on flow-sorted WT pro-B cells, and on small pre-B cells from WT, *Brwd1*^*−/−*^ and *Jk1-GAGA*^*mut*^ mice (Figure 2A–D).

We observed that some nucleosome positioning in the Jk1 region was GAGA dependent. In WT cells at the pro-B cell stage, there is no strong nucleosome structure. Then in WT small pre-B cells, that are preparing for and undergoing *Igk* recombination, a nucleosome becomes positioned just upstream of the Jk1 RSS (5’ nucleosome)(Figure 2B)^22^. However, in both *Jk1-GAGA*^*mut*^ and *Brwd1*^*−/−*^ small pre-B cells, the 5’ region that is normally occupied by a nucleosome in WT cells remained largely clear. In contrast, the 5’ nucleosome at Jk2 persisted in *Jk1-GAGA*^*mut*^ small pre-B cells (Figure 2C). In *Jk1-GAGA*^*mut*^ small pre-B cells, the Jk1 gene body (RSS and Jk gene segment, referred hereafter as the Jk1 nucleosome) tended to be obscured by a nucleosome while in *Brwd1*^*−/−*^ small pre-B cells nucleosome occupancy was pronounced (Figure 2D). From these data, we conclude that the Jk1 GAGA motif is necessary for placement of the Jk1 5’ nucleosome. In contrast, BRWD1 appears to dictate nucleosome structure throughout the Jk1 region.

While precise positioning of a 5’ nucleosome is predicted to be important for RAG2 recruitment, RSS accessibility facilitates RAG1-mediated cleavage. Therefore, we next analyzed local accessibility at Jk in WT pro-B and small pre-B cells, *Jk1-GAGA*^*mut*^ small pre-B cells and *Brwd1*^*−/−*^ small pre-B cells. Visual inspection of representative B cell populations suggested that mutation of the Jk1 GAGA motif induced large changes in small pre-B cell Jk1 accessibility including at both the RSS and Jk1 gene body (Figure 2E–F). This corresponded to decreased accessibility at the Jk1 gene body (Figure 2G) and at the Jk1 RSS (Figure 2H). Accessibility at the Jk2 gene body and RSS were not significantly affected (Figure 2I–J). These data indicate that the Jk1 GAGA motif preferentially regulates Jk1 accessibility.

Germline transcription is induced prior to *Igk* recombination and has been linked to recombination and accessibility at the *Tcra* locus^8, 35^. Therefore, we next assessed if the Jk1 GAGA motif was required for Jk germline transcription. To interrogate this, we analyzed paired-end RNA-sequencing (RNA-seq) data from WT and *Jk1-GAGA*^*mut*^ small pre-B cells. To specifically analyze germline transcription, and not transcription of recombination products, we removed multimapping reads from our analysis.

Analysis of germline Jk transcription in small pre-B cells revealed an approximately two-fold increase in Jk1 transcription while transcription of the other Jks were normal (**Supplementary Figure 3A**). This apparent increase could result from decrease recombination at Jk1 in *Jk1-GAGA*^*mut*^ small pre-B cells. As expected, Vk germline transcription was normal (**Supplementary Figure 3B**). Given that overall germline transcription was unchanged, these data suggest that the Jk1 GAGA motif does not meaningfully regulate transcription. Rather, the Jk1 GAGA motif specifically regulates the local epigenetic landscape.

### The epigenetic landscape in Rag1^−/−^IgH^+^ small pre-B cells is aberrant

Previously, *Rag1*^*−/−*^*IgH*^*+*^ mice have been a preferred model to study developmental mechanisms in small pre-B cells as this genetic construct prevents ongoing recombination which could complicate some studies. However, it is unclear if this model replicates the epigenetic state of WT small pre-B cells. Therefore, sorted WT large and small pre-B cells, and *Rag1*^*−/−*^*IgH*^*+*^ small pre-B cells, were subjected to ATAC-seq. A principal component analysis revealed *Rag1*^*−/−*^*IgH*^*+*^ small pre-B cells were intermediate between WT large and small pre-B cells on the principal component (PC) 1 axis (**Supplementary Figure 4A**). However, PC2 indicated that *Rag1*^*−/−*^*IgH*^*+*^ small pre-B cells also displayed cell specific aberrancies not attributable to developmental arrest. Indeed, the PC2 variance accounted for 35.6% of the total variance across all three populations. Visual inspection of nucleosome structure at Jk revealed several differences in *Rag1*^*−/−*^*IgH*^*+*^ small pre-B cells compared to WT small pre-B cells (**Supplementary Figure 4B**). Quantifying across mice revealed a trend towards a diminished 5’ nucleosome (**Supplementary Figure 4C**), and a prominent Jk1 nucleosome (**Supplementary Figure 4D**). Using ATAC-seq to assess accessibility (**Supplementary Figure 4E)** revealed diminished accessibility across the Jk region. Quantitation revealed trends towards less accessibility at the Jk1 gene body (**Supplementary Figure 4F**) and RSS (**Supplementary Figure 4G**) in *Rag1*^*−/−*^*IgH*^*+*^ small pre-B cells. As we have previously demonstrated that *Igk* recombination is infrequent in WT sorted small pre-B cells ^8^, we did not breed our mice to the *Rag1*^*−/−*^*IgH*^*+*^ background.

### Inhibition of Jk1 recombination impairs B lymphopoiesis

**Figure 3:**
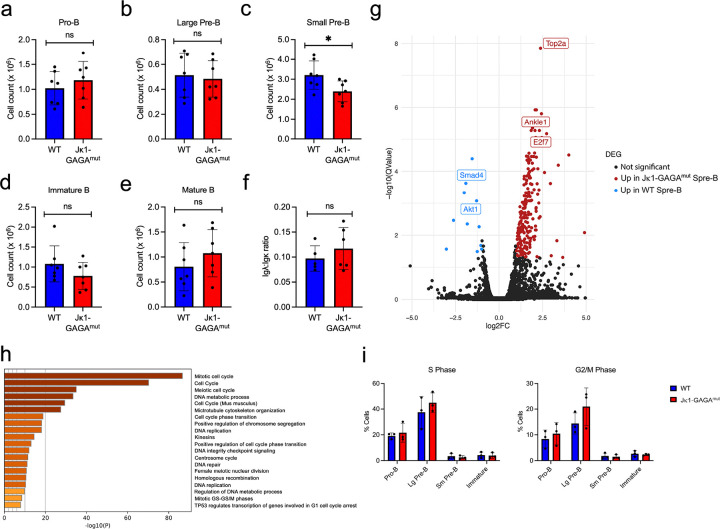
Mutation of Jk1 GAGA motif impairs B cell development. **(A-E)** Flow cytometric analysis of different developmental stages of B cell lymphopoiesis in the bone marrow of WT and *Jk1-GAGA*^*mut*^ mice. **(F)** Quantification of the Igl+ to Igk+ ratio of immature B cells of WT and *Jk1-GAGA*^*mut*^ mice. **(G)** Differential expressed genes in *Jk1-GAGA*^*mut*^ vs. WT small pre-B cells (n=2 mice). Genes upregulated in *Jk1-GAGA*^*mut*^ cells at right with down regulated genes at left. **(H)** Gene ontology analysis of differentially expressed pathways between *Jk1-GAGA*^*mut*^ and WT small pre-B cells (n=2 mice). **(I)** Cell cycle analysis in indicated bone marrow B cell populations (n=3 mice)(*p < 0.05, unpaired t-test).

We next characterized the *Jk1-GAGA*^*mut*^ mice for developmental defects by subjecting harvested BM to flow cytometry (Figure 3A–E). Small pre-B cells, were significantly reduced in *Jk1-GAGA*^*mut*^ mice as compared to WT. All other BM B cell populations were normal. By flow cytometry, the Igk/Igl ratio was similar in WT and *Jk1-GAGA*^*mut*^ in immature B cells (Figure 3F).

It was surprising that specific disruption of just Jk1 recombination would induce even a mild defect in B cell development. Therefore, we compared RNA-seq from WT and *Jk1-GAGA*^*mut*^ small pre-B cells. Indeed, in *GAGA*^*mut*^ small pre-B cells 213 genes were increased (p < 0.05), including several genes associated with proliferation including *Top2a*, *Ankle1* and *E2f7* (Figure 3G). Fewer genes were downregulated. Gene ontology analysis of differentially expressed genes between *Jk1-GAGA*^*mut*^ and WT small pre-B cells revealed general upregulation of cell division pathways (Figure 3H). This was associated with a small but statistically non-significant increase of *Jk1-GAGA*^*mut*^ large pre-B cells in S and G2/M cell cycle phases (Figure 3I). This is consistent with the mild developmental arrest observed in *Jk1-GAGA*^*mut*^ mice. Cells must fully exit large pre-B cell proliferative programs before initiating *Igk* recombination in small pre-B cells^1, 34^. These data suggest a mild defect in that transition. That inhibiting Jk1 recombination would impair exiting the large pre-B cell proliferative program is consistent with the known role of RAG-mediated DNA double-stranded breaks in inhibiting large pre-B cell proliferation^36^.

### A GAGA motif is required to rescue Jk3 recombination

**Figure 4:**
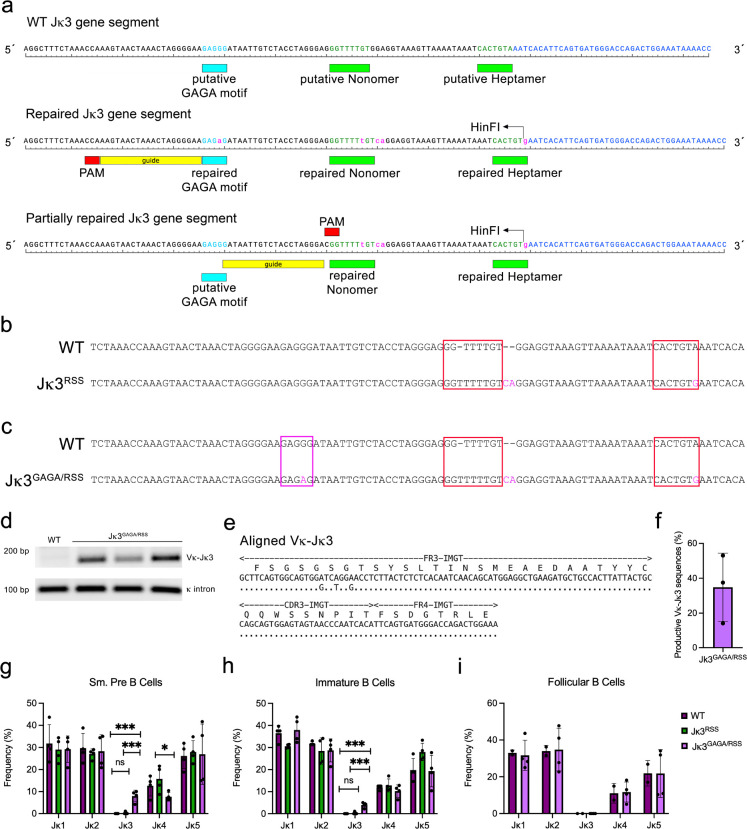
Both RSS and GAGA motifs required to rescue Vk-Jk3 recombination. (**A**) Diagram of Jk3 region with partially repaired (repaired RSS) and fully repaired (RSS and GAGA motif) sequences shown. Shown are the guides used. See methods for repair templates. Introduced *HinFI* sequence facilitated screening. (**B-C**) DNA sequence from murine founders that were then bred to homozygosity. (**D-E**) Vk-Jk3 gene product was amplified by PCR from genomic DNA (D, n=3 mice) prepared from flow sorted small pre-B cells from WT and *Jk3*^*GAGA/RSS*^ mice and individual clones sequenced (an example clone sequenced shown in E). Top line represents the observed Vk-Jk3 sequence and the bottom line represents the expected sequence. **(F)** Quantification of clones sequenced for productive and in-frame Vk-Jk3 from three individual mice. **(G-I)** Shown are the frequencies of indicated Vk-Jk gene products from WT, *Jk3*^*RSS*^ and *Jk3* ^*GAGA/RSS*^ mice from flow-sorted small pre-B (G), immature B (H) and follicular B (I) cells. Each point a separate mouse, unpaired t-test, *p <0.05, **p <0.01, ***p<0.001.

Jk3 is a pseudogene segment lacking both intact RSS and GAGA motifs. There are no pre-mature stop codons in the Jk3 gene segment and there is an intact splice donor site. Remnants of both motifs are present at Jk3 suggesting evolutionarily loss of recombination competency (Figure 4A). Therefore, we used CRISPR-CAS9 gene editing with templates designed to repair the RSS nonamer and heptamer with or without repair of the 5’ putative GAGA motif. A *HinF1* site was introduced to facilitate screening of the RSS repair. Mice were screened by PCR and sequencing. Heterozygotes were bred to homozygosity to generate Jk3^RSS^ and Jk3^GAGA/RSS^ mice (Figures 4B and C respectively).

As no validated PCR primers was available for Jk3, we designed a primer specific to the Jk3 gene body (Supplemental Table 1). Genomic DNA isolated from flow sorted small pre-B cells from WT and three Jk3^GAGA/RSS^ mice was subjected to PCR using the degenerate Vk primer and the Jk3 primer. As can be seen, a Vk-Jk3 PCR product was detected in Jk3^GAGA/RSS^ and not WT mice (Figure 4D). The resulting PCR product was then TOPO cloned and individual clones sequenced (24 clones/mouse). All sequences had high homology to Jk3 (representative sequence shown in Figure 4E). Of these clones, an average of 35% of the sequences were productive and in-frame which is consistent with stochastic recombination and lack of selection in small pre-B cells. (Figure 4F).

To look at the frequency of all Vk-Jk gene products, we sorted small pre-B cells from WT, Jk3^RSS^ and Jk3^GAGA/RSS^ mice. mRNA was isolated, copied into cDNA and PCR performed with degenerate Vk primers and a Ck primer. Individual PCR products were cloned and sequenced. All three mouse lines demonstrated similar frequencies of Vk-Jk products containing Jk1, Jk2 and Jk5. Jk3^GAGA/RSS^ had a small decrease in Jk4 containing recombination products (Figure 4G). Remarkably, 7.5% (27/358 sequences) of Jk3^GAGA/RSS^ small pre-B cells contained Vk-Jk3 expressed sequences. In contrast, only 1 of 400 sequenced PCR products (0.25%) from Jk3^RSS^ small pre-B cells contained a Vk-Jk3 product. Addition of a GAGA motif increased recombination 40-fold such that recombination frequencies to Jk3 and Jk4 were similar in Jk3^GAGA/RSS^ small pre-B cells. Therefore, both a GAGA motif and an RSS are necessary and sufficient for recombination to Jk gene segments in small pre-B cells.

We next assessed Vk-Jk3 recombination frequencies in BM immature B cells and splenic follicular B cells (Figures 4H and 4I). In immature B cells, 3.9% percent of sequences contained Vk-Jk3 products (15/384). In contrast, we detected no Vk-Jk3 expressed gene products (0/200) in follicular B cells.

**Figure 5:**
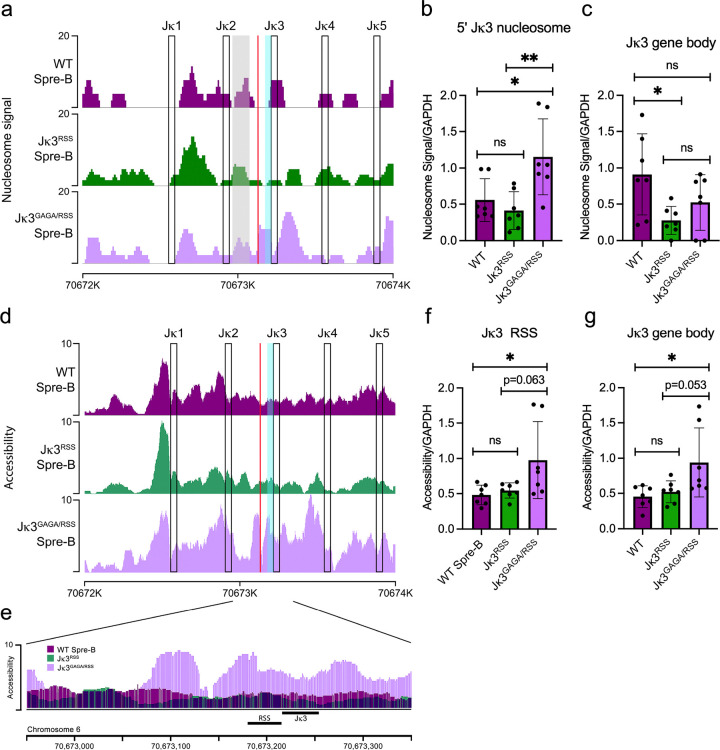
A GAGA motif restored Jk3 accessibility. **(A)** Example of nucleosome positioning at the Jk locus in small pre-B cells from WT, *Jk3*^*RSS*^ and *Jk3* ^*GAGA/RSS*^ mice. Open boxes denote the indicated gene bodies, the cyan shaded area indicates the RSS and the red line indicates the GAGA motif. Jk3 5’ nucleosome shown by grey box. Data are representative of seven independent experiments (mice). **(B)** Quantification of Jk3 5’ nucleosome. **(C)** Quantification of nucleosome signal over the Jk3 gene body. **(D)** Example of accessibility at the Jk locus in indicated mouse strains as measured by ATAC-seq. The y axis represents tags per million reads. Data representative of seven independent experiments (mice). **(E)** Example of accessibility at the Jk3 locus in the indicated mouse strains. **(F-G)** Quantification of chromatin accessibility at the Jk3 RSS (F) and Jk3 gene body(G). (each point a separate mouse, unpaired t-test, *p <0.05).

We then used ATAC-seq data to compare nucleosome positioning at Jk3 in WT small pre-B, Jk3^RSS^ small pre-B and Jk3^GAGA/RSS^ small pre-B cells. In WT small pre-B cells, Jk3 is enriched for nucleosomes including 5’ and over the Jk gene body (Figure 5A–B). The presence of a 5’ nucleosome in WT cells obscured potential contributions of the restored GAGA motif at this position. However, in Jk3^GAGA/RSS^ small pre-B cells, there was enhanced nucleosome occupancy at the 5’ position (Figure 5B). Furthermore, a distinct nucleosome over the Jk3 gene body (gene body plus RSS) was diminished in Jk3^RSS^ and tended to be decreased in Jk3^GAGA/RSS^ small pre-B cells (Figure 5C).

We then used the ATAC-seq data to examine Jk accessibility (Figure 5D–E). Representative examples revealed that addition of the GAGA and RSS 5’ to Jk3, but not the RSS alone, conferred increased accessibility across the Jk3 region. Indeed, quantitative analyses revealed that in WT small pre-B cells, Jk3 RSS and gene body gene segment accessibilities were low. Accessibility at both segments increased in Jk3^GAGA/RSS^ and not Jk3^RSS^ compared to WT small pre-B cells (Figure 5F–G). There was a trend towards increased accessibility at the Jk3 RSS and gene body in Jk3^GAGA/RSS^ compared to Jk3^RSS^ small pre-B cells. In contrast, addition of the Jk3 RSS and GAGA motifs did not change accessibility Jk1, Jk2, Jk4 and Jk5 (**Supplementary Figure 5**). These observations suggest that a primary function of a GAGA motif at Jk3 is to enhance accessibility across the RSS/Jk3 region.

To begin to understand if GAGA motifs were used at other recombination centers, we examined 5’ nucleotide sequences at the murine IgHJ (JH) and TCRaJ (Ja) gene segments (**Supplementary Figure 6**). Indeed, GAGA motifs were found 5’ to all JH segments, which, like Jk, contain 23bp RSSs. At these sites, the GAGA motifs tended to be found around 320 bp (range: 273–361 bp) 5’ to the RSS nonamer. We also found GAGA motifs 5’ to 41 of 43 Ja gene segments. The two Ja gene segments that lacked 5’ GAGA motifs, had 3’ GAGA motifs. Spacing between the Ja RSSs and the GAGA motifs was highly variable (9-980bp). These data suggest that GAGA motifs are present at recombination centers other than Jk.

## Discussion

Herein, we demonstrate that the canonical Jk RSSs are not sufficient to ensure efficient RAG-mediated genomic cleavage and recombination. Rather, a local 5’ GAGA motif is essential for efficient recombination to the Jk RSSs and for dictating local nucleosome structure and genomic accessibility. While traditionally the focus has been on the RAG cleavage site encoded by the RSS, we now demonstrate the importance of associated DNA GAGA motifs which ensure the Jk epigenetic state required for Vk-Jk recombination.

Our data support a model in which the presence of a 5’ GAGA motif ensures the epigenetic landscape necessary for efficient recombination in two ways. First, the GAGA motif ensures that the RSS is accessible, which *in vitro* studies have shown is required for permitting RAG1-mediated RSS cleavage^23,24^. Second, the GAGA motif directs positioning of a nucleosome 5’ to the RSS, which is predicted to enable precise recruitment of RAG2^19, 20, 21^. While, theoretically, a nucleosome could be positioned 3’ to the Jk gene segment, our mutational analysis at Jk1 indicates that 5’ nucleosome placement is associated with efficient recombination. Therefore, our findings indicate an expanded definition of the minimal motif necessary at Jk for Vk-Jk RAG-mediated recombination.

Our experiments demonstrated that a 5’ GAGA motif was required for efficient recombination to both Jk1 and a reconstituted Jk3. The effect of each GAGA motif, on both nucleosome placement and recombination, was primarily restricted to the proximate Jk gene segment. Furthermore, we observed that all JH gene segments also had 5’ GAGA motifs spaced similarly to each other in front of their respective RSS. In contrast to the stereotypical GAGA motifs observed at these 23bp-RSS recombination centers, the spacing at the 12bp-RSS Jα recombination center was highly variable including two of 43 Jα gene segments having 3’ GAGA motifs. These latter observations suggest the rules governing recombination at Jα are more permissive and/or that recombination mechanisms at some antigen receptor loci are different.

Data from the Jk1 mutant mice show a clear dependency of 5’ nucleosome positioning on GAGA motifs. It has been previously shown that BRWD1 is recruited to the Jk locus and plays an important role in both clearing local RSSs of nucleosomes and 5’ nucleosome placement^26^. Furthermore, nucleosome positioning in Jk1 GAGA-mutant small pre-B cell cells resembled that of the *Brwd1*^*−/*−^ cells at the Jk1 locus. BRWD1 is recruited genome-wide to specific epigenetic landscapes which do or do not contain extended GAGA motifs. Therefore, GAGA motifs are not required for BRWD1 genome recruitment. However, BRWD1-dependent nucleosome remodeling occurs only when GAGA motifs are present^26^. Thus, the overall picture is most consistent with GAGA Jk motifs serving as a guide for nucleosome positioning by BRWD1. This is consistent with the role GAGA motifs play in chromatin remodeling in *Drosophila*^31^.

It is interesting that Jk3 expressing B cell progenitors were progressively depleted during B cell maturation. We observed 7.5% of small pre-B expressing Vk-Jk3 recombination products with approximately one-third of recombination products being in-frame. These observations are consistent with stochastic recombination without selection. However, in mature B cell populations, no expressed Jk3 containing Igκs were detected. Autoreactivity is purged from cells transiting from the small pre-B cell to mature B cell stages^37, 38, 39^. The observation that Jk3 expressing cells were deleted across this transition strongly suggests that Jk3 conferred autoreactivity. It is remarkable that Jk3 appeared to confer autoreactivity regardless of which Vk it recombined or which heavy chain it was paired with.

Our findings reveal that an extended recombination motif at Jk, including both the RSS and 5’ GAGA motifs, is critical for gene recombination. While the RSS encodes the necessary cleavage site for the RAG recombinase, the 5’ GAGA motif dictates the local chromatin architecture required for efficient recombination. These findings likely have implications for other recombination events involving RAG recruitment. Indeed, we found GAGA motifs 5’ to both JH and Jα RSSs suggesting a general requirement in antigen receptor gene recombination. Therefore, the GAGA motif-dependent mechanism we described in small pre-B cells, might ensure the fidelity and contextual appropriateness of RAG-mediated gene recombination in other lymphocyte populations.

## Materials and Methods

### Mice

Wild-type (C57BL/6), *Jk1-GAGA*^*mut*^ (C57BL/6), *Jk3*^*RSS*^ (C57BL/6), *Jk3*^*GAGA/RSS*^ (C57BL/6) and *Brwd1*^−/−^ (C57BL/6-C3HeB/FeJ) mice were housed in the animal facility of the University of Chicago. Male and female mice were used at 6–12 weeks of age in accordance with the Institutional Animal Care and Use Committee of the University of Chicago.

### CRISPR-Cas9 Gene Editing

CRISPR-Cas9 RNAs (crRNAs) were designed using the crRNA design resource hosted by the Feng Zhang lab and CHOPCHOP (Supplementary Table 1)^40, 41^. crRNAs were optimized for high efficiency and low off-target scores and were synthesized by Integrated DNA Technologies (IDT) as Alt-R CRISPR-Cas9 crRNA oligos. To produce the guide RNA (gRNA), crRNA and tracrRNA (IDT) were resuspended at 1 μg/μl in injection buffer (1 mM Tris-HCl, pH 7.5, 0.1 mM EDTA), mixed at a 1:2 ratio by mass and annealed in a thermocycler (95 °C for 5 min, ramp down to 25 °C at 5 °C/min).

To produce the active ribonucleoprotein (RNP) mixture, a 100 μl solution was prepared containing the gRNA and Alt-R S.p. Cas9 Nuclease (IDT). For the *Jk1-GAGA*^*mut*^, *Jk3*^*RSS*^ *and Jk3* ^*GAGA/RSS*^ mice, 300 ng/μl of gRNA and 300 ng/μl of Cas9 was used as well as 100 ng/μl of Cas9 mRNA. The RNP mix was incubated at room temperature for 15 min after which 200 ng/μl of ssODN repair template was added. The RNP mix was then centrifuged at 13,000 RPM at room temperature to remove solid particles, and the top 80 μl was used. Injections were performed by the University of Chicago Transgenics Core. One cell fertilized embryos were injected with the RNP mix and implanted into pseudo-pregnant mice. Due to the heterogeneous nature of CRISPR-Cas9 gene editing, both alleles were screened for edits separately in the F0 generation. The region surrounding the targeted DNA was amplified by PCR using primers listed in Supplementary Table 1, and products were cloned into a TA vector. Genotyping vectors were transformed and a minimum of 8 individual colonies were sequenced.

### ATAC-seq

ATAC-seq was performed with 1.2 × 10^5^ FACS-sorted small pre-B and immature B cells as previously described^26, 42^. Briefly, cells were washed with PBS and lysed with ATAC lysis buffer (10 mM Tris-HCl, pH 7.4, 10 mM NaCl, 3 mM MgCl2, 0.1% IGEPAL CA-630). Nuclei were incubated with the transposase tagmentation enzyme (Illumina). Library fragments were amplified using the Nextera Indexing kit (Illumina) and NEBNext PCR master mix (New England BioLabs) for 10–12 cycles and were purified with the QIAquick PCR Purification Kit (Qiagen). Libraries were size-selected with the E-Gel SizeSelect gel system (Life Technologies) in the range of 150–650 bp. We quantified the size-selected libraries with an Agilent Bioanalyzer and via qPCR in triplicate using the KAPA Library Quantification Kit on the Life Technologies Step One System. Libraries were sequenced on the Illumina HiSeq2000.

ATAC-seq data was analyzed as previously described^26, 42^. For comparative analysis of chromatin accessibility between samples, we used the bedtools bigWigAverageOverBed, with the open chromatin bigWig file and a bed file containing annotated regions of interest as inputs. Samples were normalized to accessibility at *Gapdh*. Similarly, for comparative analysis of nucleosome occupancy between samples, we instead used the nucleosome signal bigWig file.

### Quantitative PCR, RNA-seq, and analysis

Total cellular RNA was isolated with a RNeasy kit (Qiagen) and RNA was reverse transcribed with SuperScript III reverse transcriptase (Invitrogen). A total volume of 25 μl containing 1 μl cDNA template, 0.5 μM of each primer (Supplementary Table 1) and PowerUp SYBR Green PCR Master Mix (Applied Biosystems) was analyzed in triplicate. Gene expression was analyzed with an ABI QuantStudio 3 and ABI QuantStudio Design & Analysis Software v1.5.2 (Applied Biosystems). Normalized results were calculated using the ΔΔCt method ($\text{Expression}\;\text{Fold}\;\text{Change}\;=2^{\wedge }-\left(\left({\text{KO}}_{\text{TEST}}-{\text{KO}}_{\text{HOUSEKEEPING}}\right)-\left({\text{WT}}_{\text{TEST}}-\right.\right.\left.{\text{WT}}_{\text{HOUSEKEEPING}}\right))$ using *B2m* as the housekeeping gene.

For RNA-seq, RNA libraries were prepared using the standard Illumina library protocol (Kit, RS-122–2101 TruSeq Stranded mRNA LT-SetA) before sequencing on the Illumina HiSeq2500. Raw reads were aligned to reference genome mm9 in a splice-aware manner using STAR^43^. Gene expression was quantified using FeatureCounts against UCSC genes, with Ensembl IG genes from mm10 converted to mm9 coordinates with UCSC liftOver^44^.

Differential expression statistics (fold-change and p value) were computed using edgeR on raw expression counts obtained from quantification^45^. Pairwise comparisons were computed using exactTest, and multigroup comparisons using the generalized linear modeling capability in edgeR. In all cases, p values were adjusted for multiple testing using the FDR Benjamini-Hochberg correction. Significant genes were determined based on an FDR threshold of 0.05 in the multigroup comparison. Metascape was used for pathway analyses^46^.

#### PCR analysis of Igk rearrangements

Quantification of Jk usage by PCR was performed by sequencing the products of *Igk* rearrangements. Degenerate Vk and Ck primers were used along with 2 μl of cDNA in a 25 μl reaction using Platinum Taq DNA polymerase (ThermoFisher). Two μl of the PCR product was cloned into the pCR2.1-TOPO TA vector (ThermoFisher) and transformed into DH5α cells (ThermoFisher). DNA was extracted from resulting colonies with the QIAprep Spin Miniprep Kit (Qiagen) and sequenced. Unique sequences were analyzed for Jk usage by alignment to the mouse Jk (Supplementary Table 1) and Vk sequences using the NCBI IgBLAST alignment tool (https://blast.ncbi.nlm.nih.gov/).

### Flow cytometry and flow activated cell sorting (FACS)

Bone marrow was extracted from the hind leg bones of mice, suspended in RPMI media with 10% vol/vol FBS, and passed through a 70 μm filter. Red blood cells were lysed with ACK lysis buffer (Lonza). Cells were then washed and resuspended in staining buffer (PBS with 3% vol/vol FBS) and blocked with 2.5 μl of Fc block (BD Pharmingen) for 10 min. Cells were stained with anti-B220-PE/Cy7 (RA3–6B2), anti-CD19-PerCP/Cy5.5 (1D3), anti-CD43-PE (S7), anti-IgM-APC (II/41), anti-Igk-BV510 (187.1), anti-Igλ-FITC (R26–46). Small pre-B cells (B220^+^CD43^−^IgM^−^FSC^low^) and immature B cells (B220^+^CD43^−^IgM^+^) were isolated by cell sorting with a FACSAriaII (BD). Flow cytometric analysis was done with FlowJo (BD).

#### Analysis of Igk rearrangements on genomic DNA

Genomic DNA was isolated from flow sorted small pre-B cells from WT and *Jk3*^*GAGA/RSS*^ mice as described^26^. PCR was performed using the deg Vk and a Jk3 specific primer (Supplementary Table 1) on 50 ng of template DNA. A region in Eki was amplified to control for the amount of genomic DNA (primers Eki-F and Eki-R). The resulting PCR product was cloned into pCR2.1-TOPO TA (Invitrogen) and individual colonies sequenced and aligned using the NCBI IgBLAST alignment tool (https://blast.ncbi.nlm.nih.gov/).

### Statistical analysis

Statistical analyses were performed with GraphPad Prism. For multiple comparisons, data were analyzed by analysis of variance in combination with Tukey’s multiple comparisons test. Bar graphs are displayed as the mean ± S.E.M. Significance as defined by *P* value or FDR are provided in the figures, figure legends or in corresponding text. Additional quantitative methods and statistical criteria are mentioned above based on their respective technology and analysis approaches.

## Supplementary Material

Supplementary Files

This is a list of supplementary files associated with this preprint. Click to download.

• Supp.Fig.1.tif

• Supp.Fig.2.tif

• Supp.Fig.3.tif

• Supp.Fig.4.tif

• Supp.Fig.5.tif

• Supp.Fig.6.tif

• SupplementaryTable1.docx

## Data Availability

Gene Expression Omnibus accession codes for our publicly available datasets are as follows: GSE173652 (ATAC-seq and RNA-seq for WT pro-B and WT small pre-B cells); GSE288586 (ATAC-seq for WT, Jk1-GAGA^mut^, Jk3^RSS^ and Jk3 ^GAGA/RSS^ small pre-B cells as well as RNA-seq for WT and Jk1-GAGA^mut^ small pre-B cells). The reviewer access token for GSE288586 is qpgfieewhlyrhat.
